# Knigth's Move in the Periodic Table, From Copper to Platinum, Novel Antitumor Mixed Chelate Copper Compounds, Casiopeinas, Evaluated by an *in Vitro* Human and Murine Cancer Cell Line Panel

**DOI:** 10.1155/MBD.2001.19

**Published:** 2001

**Authors:** Isabel Gracia-Mora, Lena Ruiz-Ramírez, Celedonio Gómez-Ruiz, Mabel Tinoco-Méndez, Adriana Márquez-Quiñones, Laura Romero-De Lira, Álvaro Marín-Hernández, Lucía Macías-Rosales, Ma. Elena Bravo-Gómez

**Affiliations:** 1 Inorganic Chemistry Department Faculty of Chemistry, UNAM Mexico City Z.P. 04510 Mexico; 2 Animal Experimentation Unit Faculty of Chemistry, UNAM Z.P. 04510 Mexico City Mexico; 3 National Cancer Institute-Mexico Mexico City Mexico

## Abstract

We synthesized a novel anticancer agents based on mixed chelate copper (II) complexes, named Casiopeínas^®^ has of general formula [Cu(N-N)(N-O)H_2_O]NO_3_ (where, N-N = diimines as 1,10- phenanthroline, 2,2-bipyridine, or substituted and N-O=aminoeidate or [Cu(N-N)(O-O)H_2_O]NO_3_ (where NN= diimines as 10-phenanthroline, 2,2-bipyridine or substituted Casiopeínas I, II, IV, V, VI, VII VIII and O-O=acetylacetonate, salicylaldehidate Casiopínas III). We evaluated the in vitro antitumor activity using a human cancer cell panel and some nurine cancer cells. Eleven Casiopeinas are evaluated in order to acquire some structure-activity correlations and some monodentated Casiopeinäs analogues; cisplatinum was used as control drug. The 50% growth inhibition observed is, in all cases reach with concentrations of Casiopeina's 10 or 100 times lower than cisplatinum. In a previous work we reported the induction of apoptosis by Casiopeina II. The results indicate that Casiopeinass are a promising new anticancer drug candidates to be developed further toward clinical trials.


### KNIGTH'S MOVE IN THE PERIODIC TABLE, FROM COPPER TO PLATINUM, NOVEL ANTITUMOR MIXED CHELATE COPPER COMPOUNDS, CASIOPEINAS, EVALUATED BY AN *IN VITRO* HUMAN AND MURINE CANCER CELL LINE PANEL

Isabel Gracia-Mora[1,2], Lena Ruiz-Ramírez*[1], Celedonio Gómez-Ruiz[2,3], Mabel Tinoco-Méndez[2], Adriana Márquez-Quiñones[1], Laura Romero-De Lira[1], Álvaro Marín-Hernández[1], Lucía Macías-Rosales[2], Ma. Elena Bravo-Gómez[1]

[1] Inorganic Chemistry Department, Faculty of Chemistry, UNAM, Z.P. 04510, Mexico City, Mexico
[2] Animal Experimentation Unit, Faculty of Chemistry, UNAM, Z.P. 04510, Mexico City, Mexico
[3] National Cancer Institute-Mexico, Mexico City, Mexico

### ABSTRACT

We synthesized a novel anticancer agents based on mixed chelate copper (II) complexes, named Casiopeínas® has of general formula $[Cu(N\text{-}N)(N\text{-}O)H_2O]NO_3$ (where, N-N= diimines as 1,10-phenanthroline, 2,2-bipyridine, or substituted and N-O=aminocidate or $[Cu(N\text{-}N)(O\text{-}O)H_2O]NO_3$ (where N-N=diimines as 1,10-phenanthroline, 2,2-bipyridine or substituted Casiopeinas I, II, IV, V, VI, VII VIII and O-O=acetylacetonate, salicylaldehidate Casiopeínas III). We evaluated the in vitro antitumor activity using a human cancer cell panel and some nurine cancer cells. Eleven Casiopeinas are evaluated in order to acquire some structure-activity correlations and some monodentated Casiopeina¨s analogues; cisplatinum was used as control drug. The 50% growth inhibition observed is, in all cases reach with concentrations of Casiopeina's 10 or 100 times lower than cisplatinum . In a previous work we reported the induction of apoptosis by Casiopeina II. The results indicate that Casiopeinass are a promising new anticancer drug candidates to be developed further toward clinical trials.

### INTRODUCTION

A series of Cu (II) mixed chelate compounds Casiopeínas® has been synthesized, characterized, patented (1, 2, 3, 4) and X-ray structures solved when proper crystals were obtained (5), stability constants determined and EPR study has been done (6).

The general formula of Casiopeínas is $[Cu(N\text{-}N)(N\text{-}O)H_2O]NO_3$ (where, N-N= diimines as 1,10-phenanthroline, 2,2-bipyridine, or substituted and N-O=aminocidate or $[Cu(N\text{-}N)(O\text{-}O)H_2O]NO_3$ (where, N-N=diimines as 1,10-phenanthroline, 2,2-bipyridine or substituted Casiopeinas I, II, IV, V, VI, VII VIII and O-O=acetylacetonate, salicylaldehidate Casiopeínas III). The design of the molecules was based in three main factors: the compounds should contain an essential metal for diminish toxicity; contain chelates that favor the cis-configuration around the metal ion and the mixed chelates that contain different level of hydrophobicity. Casiopeínas were design to have antitumor activity, based on previous works in cisplatinum and other transition metal series. These reported compounds are proposed to present some degree of DNA-interaction.

A preliminary report of antineoplastic activity was presented (7), also SOD like activity and the induction of apoptosis has been reported (8, 9). Casiopeínas have shown cytotoxicity in several murine tumoral cell lines and strong *in vivo* antitumor activity in murine tumoral models in our preliminar results (10).

The present study was designed to evaluate the in vitro antitumor activity of several Casiopeínas against various human and murine tumoral cell lines and to observe a correlation of activity as a function of the peripherical substituents on the ligands. The *in vitro* test is one of the most adequate methods to evaluate anticancer activity in a large range of compounds. In Figure 1 the structure of Casiopeina III-I is shown as the perchlorate salt (6).

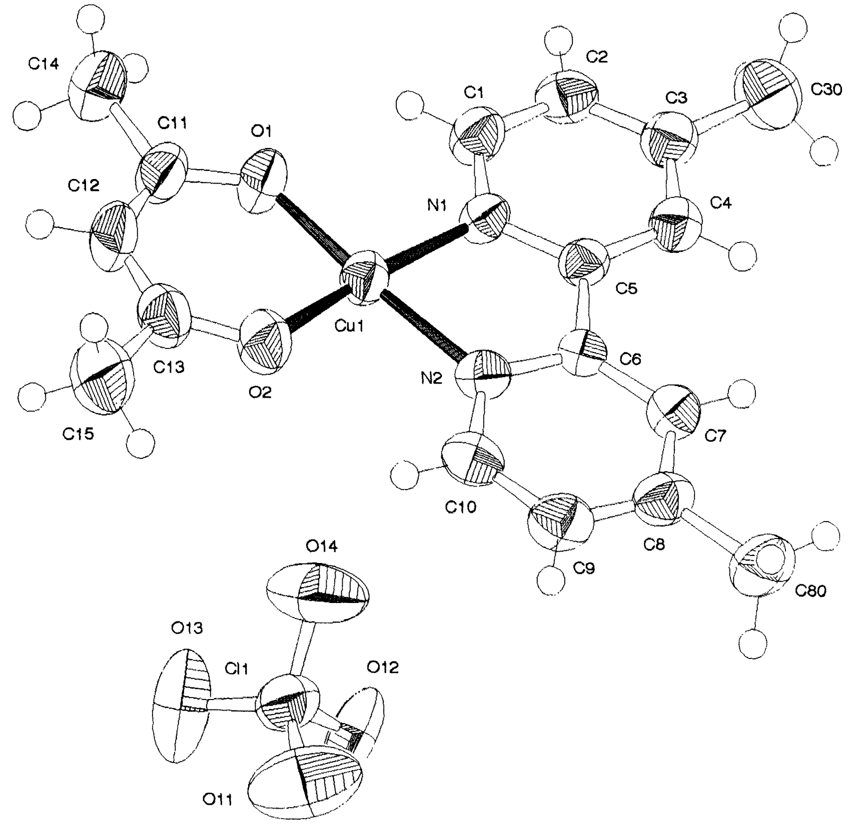

Figure 1. Structure of Casiopeina III-I, (4,4-dimethyl, 2,2-bipyridine) (acetylacetonate) copper (II) perchorate (6)

**MATERIALS AND METHOD**

CASIOPEINAS® were synthesized following the methodology reported in the Patents (1). Equimolecular solution of the $CuNO_3$ and the corresponding diimine are mixed together followed by an equimolecular aqueous solution of the charged ligand, previously deprotonated. The resulting solution is concentrated and the solid obtained is filtered and recristallized several times.

Studied Drugs

Casiopeína Igly- Aqua (4,7-diphenyl-1,10-phenanthroline) (glycine) copper (II) Nitrate.

Casiopeína Iser- Aqua (4,7-diphenyl-1,10-phenanthroline) (serine) copper (II) Nitrate.

Casiopeína IIgly- Aqua (4,7-dimethyl-1,10-phenanthroline) (glycine) copper (II) Nitrate.

Casiopeína IIgly- Aqua (4,7-dimethyl-1,10-phenanthroline) (serine) copper (II) Nitrate.

Casiopeína III-I- (4,4-dimethyl, 2,2-bipyridine) (acetylacetonate) copper (II) Nitrate.

Casiopeína III-E- (4,7-dimethyl-1,10-phenanthroline) (acetylacetonate) copper (II) Nitrate.

Casiopeína IVgly- Aqua (4,4-dimethyl, 2,2-bipyridine) (glycine) copper (II) Nitrate.

Casiopeína Vgly- Aqua (5-nitro-1,10-phenanthroline) (glycine) copper (II) Nitrate.

Casiopeína III 5acac- Aqua (5-nitro-1,10-phenanthroline) (acetylacetonate) copper (II) Nitrate.

Casiopeína Vser- Aqua (5-nitro-1,10-phenanthroline) (serine) copper (II) Nitrate.

Control drug

CDDP-Cis-diammine-dichloro-platinum (II)

Monochelates copper compounds
Copper Nitrate
129Bis Aqua (4,7-diphenyl, 1,10-phenanthroline) copper (II) Nitrate.
128Bis Aqua (4,7-dimethyl, 1,10-phenanthroline) copper (II) Nitrate.

133Bis Aqua (5-nitro-1,10-phenanthroline) copper (II) Nitrate.
131Bis Aqua (4,4-dimethyl, 2,2-bipyridine) copper (II) Nitrate
134Bis Aqua (glycine) copper (II) Nitrate
135Bis Aqua (acetylacetonate) copper (II) Nitrate

All starting materials and CDDP were commercial and used without further purification.

**CELL LINES**

Cancer Cell Line Panel. To evaluate drugs for the cell growth inhibition profile, we established a human cancer cell line or murine tumoral cell line from the panel described (11). With this system we have examined the antiproliferative effect of the Casiopeínas, and basal control drugs mentioned above.

Human: HeLa (adenocarcinoma Stage IV A), SiHa (carcinoma Stage IIIB), CaSki (carcinoma Stage II B), C33-A (carcinoma Stage III A), were obtained from the American Type Culture Collection and CaLo (carcinoma Stage II B) and InBl were cloned from biopsies of patients from the National Cancer Institute-Mexico and generously donated for this project (12).

Murine: B16 Melanoma and Lewis Lung Carcinoma were obtained from the American Type Culture Collection (12).

Measurements of Cell Growth Inhibition. From a stock culture cell obtained from each cell line cultured in Dulbecco's Modified Eagle Medium (DMEM) supplemented with 10% of fetal bovine serum (FBS), at 37 °C in humidified air containing 5 % $CO_2$, a cell dilution of $10^6$ cells/ml is prepared. From this solution, a volume of 20 μl is added to each well of the microplate in order to obtain $2*10^4$ cells/well. Previously, each well must contain 100 μl of RPMI 1640 and 10% FBS, then the cells are incubated at 37° C and 5 % of $CO_2$ for 24 hours. This procedure is in order to allow the cells to attach to the bottom of the well. After the 24 hours, the cells are already attached, and the medium and FBS are vacuumed from the wells and then added with 90 μl of DMEM with 10% of FBS and 10 μl of the four different concentrations of the drugs (Casiopeinas ), basal control drugs or CDDP: 100 μg/ml, 10 μg/ml, 1 μg/m and 0.1 μg/ml, one well is not added with drugs and it is the proliferation control. Then are incubated for 24 hours. After the time of incubation, the medium is vacuumed, the cells are fixed with 200 μl of trichloroacetic acid at 10 % over 1hour at 4°C. Finally the cells are washed 5 times with regular water and left to dry at room temperature and then stained with 100 μl of sulforhodamine-B at 0.4% to each one of the wells containing the cells and incubated for 30 min at room temperature. Then are washed 4 times with acetic acid at 1%, eliminating it after been washed, are left to dry at room temperature. When the stain has been incorporated to the cells, it is solubilized with 100 μl of tris base 10mM (pH 10.5) during 5 minutes with stirring. Finally the stained cells are readed at 564 nm. Each test is repeated three times (13, 14).
The details of measuring cell growth inhibition are described elsewhere (citas26 del articulo). Briefly, the cells were plated at proper density in 96-well plates with DMEM and 10% of FBS and allow to attach for 24 hr.. The cells were exposed to drugs (Casiopeinas, Blanks and control drug) for 24 hr. Then the cell growth was determined according to the sulforhodamine B assay, described by Skehan (28del articulo). Data calculations were made according to the method described previously (26 de art). Absorbance for the control well (C) and the test well (T) were measured at 564 nm. Moreover, at time 0 (addition of the drugs), absorbance for the test well ($T_0$) was also measured. Using these measurements, cell growth inhibition (percentage of growth) by each concentration of drug was calculated as: % growth =100 X $[(T- T_0)/(C- T_0)]$, when $T > T_0$ and % growth=100 X $[(T- T_0)/ T]$, when $T < T_0$. By using the computer to process % growth values, the 50% growth inhibition parameter ($GI_{50}$ ) was determined. The $GI_{50}$ was calculated as 100 X $[(T- T_0)/(C- T_0)] = 50$ (15).

**RESULTS AND DISCUSSION**

The general project for developing new drugs is showed in the flow diagram Diagram I. In this work we present the *in vitro* evaluation over Human and Murine Tumoral Cell Lines.

The Casiopeinas chosen for this study are those that are selection of the different diimines with the porpuse of observe the effect of the same charge ligand , as glycine with several diimines, then we kept the same aminoacidate and observe the effect of the diimine. Also we have synthesized and tested some monochelates copper complexes in order to observe if the dissociated complex may present some activity.
The results on GI50 (Growth Inhibition) produced by the several Casiopeinas over the Uterine Cervix Human Tumoral Lines are shown in Table I.

Table 1

| Casiopeína | HeLa | | Siha | | Ca Ski | | C33 | | CaLo | | InBl | |
|---|---|---|---|---|---|---|---|---|---|---|---|---|
| | μg/ml | mM | μg/ml | mM | μg/ml | mM | | μg/ml | mM | μg/ml | mM | μg/ml |
| I-gly | 0.47 | 9.9E-7 | 0.4 | 8.5E-5 | 0.51 | 1.1E-6 | I-gly | 0.47 | 9.9E-7 | 0.4 | 8.5E-5 | 0.51 |
| II-gly | 0.33 | 9.5E-7 | 0.46 | 1.6E-6 | 0.42 | 1.2E-6 | II-gly | 0.33 | 9.5E-7 | 0.46 | 1.6E-6 | 0.42 |
| I-ser | 0.83 | 1.5E-3 | 6.02 | 1.1E-2 | 0.13 | 2.2E-4 | I-ser | 0.83 | 1.5E-3 | 6.02 | 1.1E-2 | 0.13 |
| II-ser | 1.22 | 2.8E-3 | 0.81 | 1.9E-3 | 0.53 | 1.2E-3 | II-ser | 1.22 | 2.8E-3 | 0.81 | 1.9E-3 | 0.53 |
| III-I | 16.84 | 4.4 E-2 | 0.01 | 2.6 E-5 | - | - | III-I | 16.84 | 4.4 E-2 | 0.01 | 2.6 E-5 | - |
| III-E | 35.83 | 9.6 E-2 | 0.15 | 4.0 E-4 | - | - | III-E | 35.83 | 9.6 E-2 | 0.15 | 4.0 E-4 | - |
| Ivgly | 34.65 | 9.0 E-2 | 4.9E-3 | 1.3 E-5 | - | - | Ivgly | 34.65 | 9.0 E-2 | 4.9E-3 | 1.3 E-5 | - |
| Vnitro (ser) | 1274.69 | 3.56 | 0.07 | 1.9 E-4 | - | - | Vnitro (ser) | 1274.69 | 3.56 | 0.07 | 1.9 E-4 | - |
| IIInitro(acac) | 6.05 | 1.3E-2 | 6.55 | 1.4E-2 | 1.49 | 3.3E-3 | IIInitro (acac) | 6.05 | 1.3E-2 | 6.55 | 1.4E-2 | 1.49 |
| Vnitro (gly) | 1.27 | 3.2E-3 | 2.45 | 6.2E-3 | 2.56 | 6.5E-3 | Vnitro (gly) | 1.27 | 3.2E-3 | 2.45 | 6.2E-3 | 2.56 |
| Cisplatin | 11522.9 | 35.02 | 1.49 | 4.5E-3 | 202.46 | 0.6155 | Cisplatin | 11522.9 | 35.02 | 1.49 | 4.5E-3 | 202.46 |

DIAGRAM 1. Flow diagram for the development of Casiopeinas.

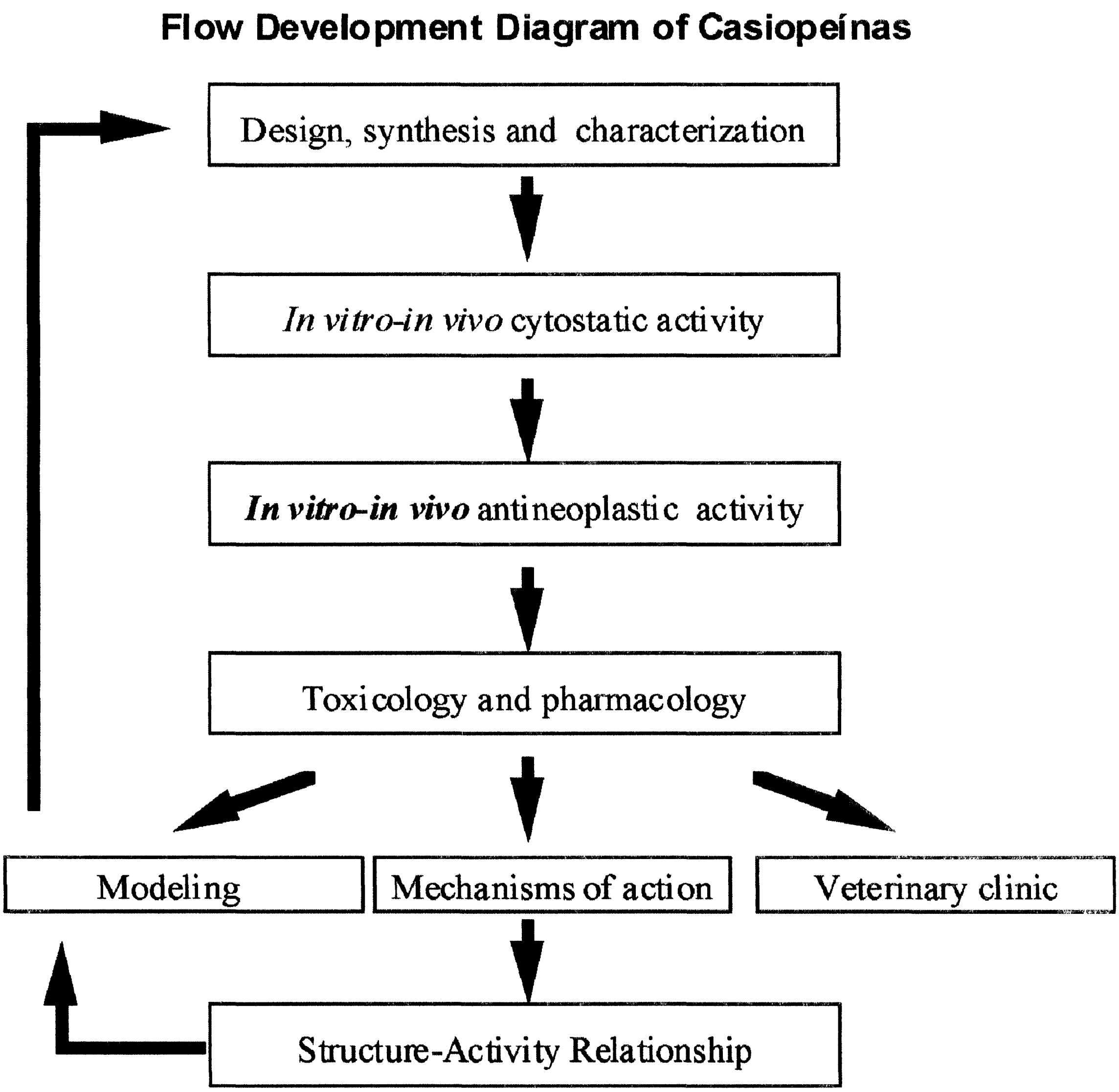

The results from Table I are shown in Plot 1

Table 2

| Casiopeína | B16 Melanoma | | Lewis Lung Carcinoma | |
|---|---|---|---|---|
| | μg/ml | mM | μg/ml | mM |
| III-I | 10761.5 | 28.13 | 1988.5E5 | 5.19E5 |
| III-E | 35.83 | 9.6E-2 | 51.68 | 7.16 |
| IVgly | 126.33 | 0.33 | 285.38 | 0.74 |
| Vnitro-ser | 10960 | 30.7 | - | - |
| II-gly | - | - | 1.25 | 0.03399 |
| II-ser | - | - | 1.055 | 0.02666 |
| Vnitro gly | - | - | 1.890 | 0.04775 |
| Cisplatin | 55.71 | 0.18 | 188.24 | 0.63 |

The results on GI50 (Growth Inhibition) produced by the several Casiopeinas over the Murine Tumoral Cell Lines are shown in Table II. These results are depicted in plot 2

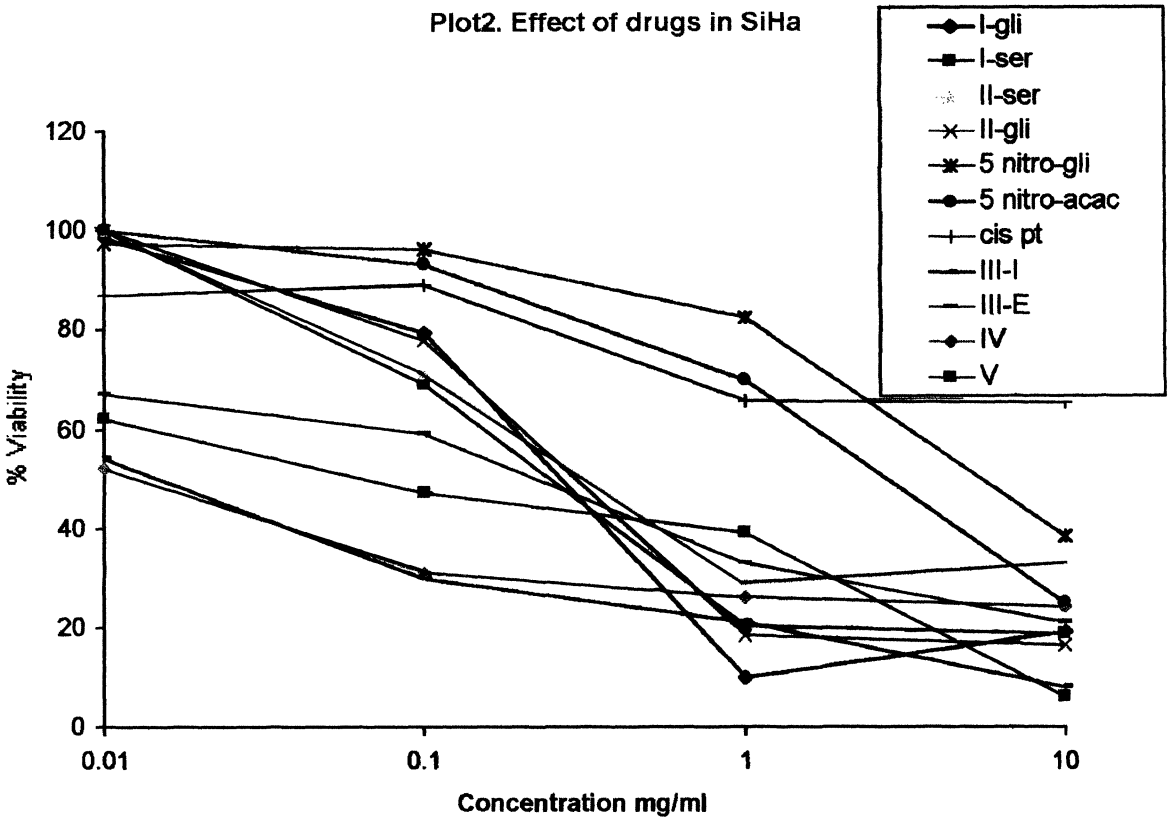

Table 3

| Basal control | GI 50 (μg/ml) HeLa | GI 50 (μg/ml) SiHa | GI 50 (μg/ml) LL Carcinoma |
|---|---|---|---|
| 128 Bis Aqua (4,7-dimethyl, 1,10-phenanthroline) copper (II) Nitrate. | α* | 10.80* | α* |
| 129 Bis Aqua (4,7-diphenyl, 1,10-phenanthroline) copper (II) Nitrate | 121.4* | α* | 288.98* / 3.29** |
| 130 | 1315.02* | α* | 41496.91* |
| 131 Bis Aqua (4,4-dimethyl, 2,2-bipyridine) copper (II) Nitrate | 26.01* | α* | 48.72v |
| 132 | 190.25* | 197.63* | 32.91* |
| 133 Bis Aqua (5-nitro-1,10-phenanthroline) copper (II) Nitrate. | 106.66* | 24.93* | 3.2* |
| 134 Bis Aqua (glycine) copper (II) Nitrate | α* | α* | α** |
| 135Bis Aqua (acetylacetonate) copper (II) Nitrate | - | 679.1** | α** |

* Dissolved in water. ** **Dissolved in DMSO.**

The % of Inhibition for each Cell Line are shown in Plots 3-8 are shown the results for the differents tumoral cell lines.

The results in GI50 (Growth Inhibition) produced by the Basal Controls over Human and Murine Tumoral Cell Lines are shown in Table III. For these basal controls the Human Tumoral Cell Lines used were HeLa and SiHa and the Murine Tumoral Cell Lines was Lewis Lung Carcinoma.

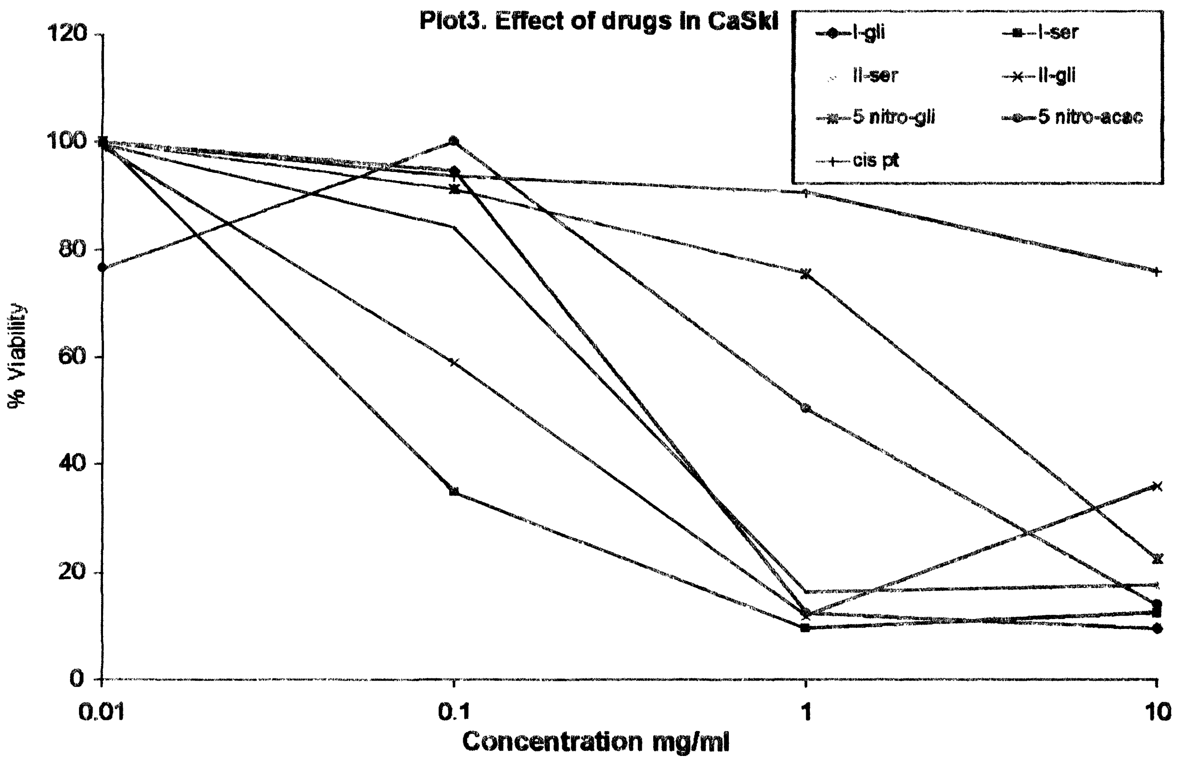

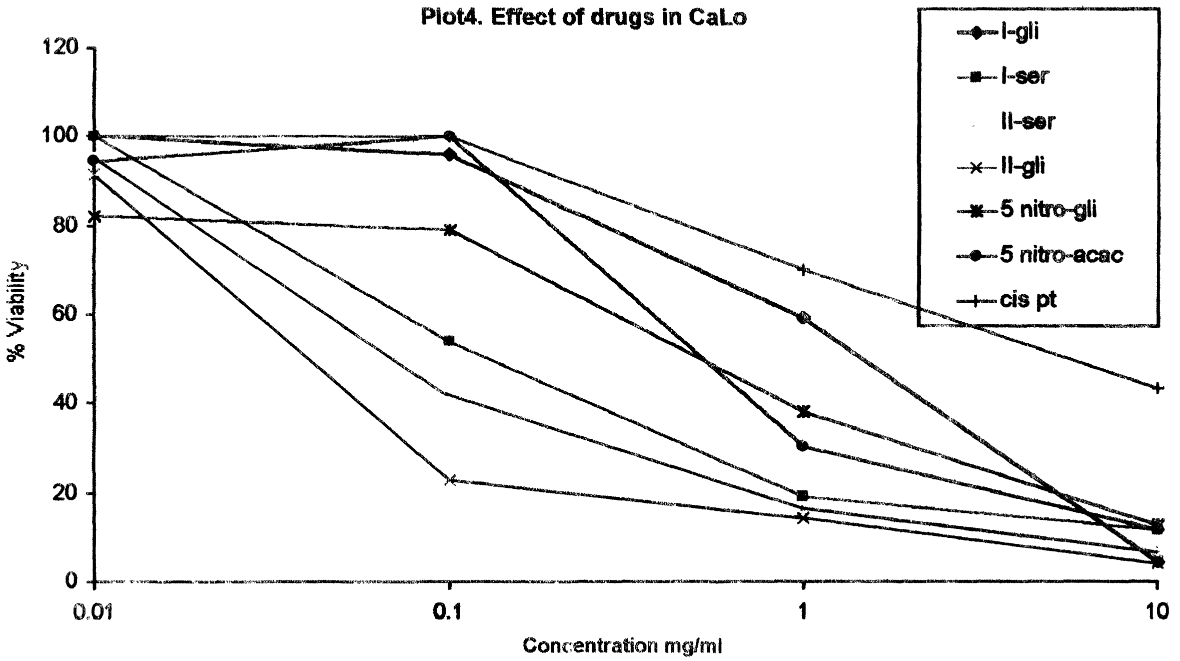

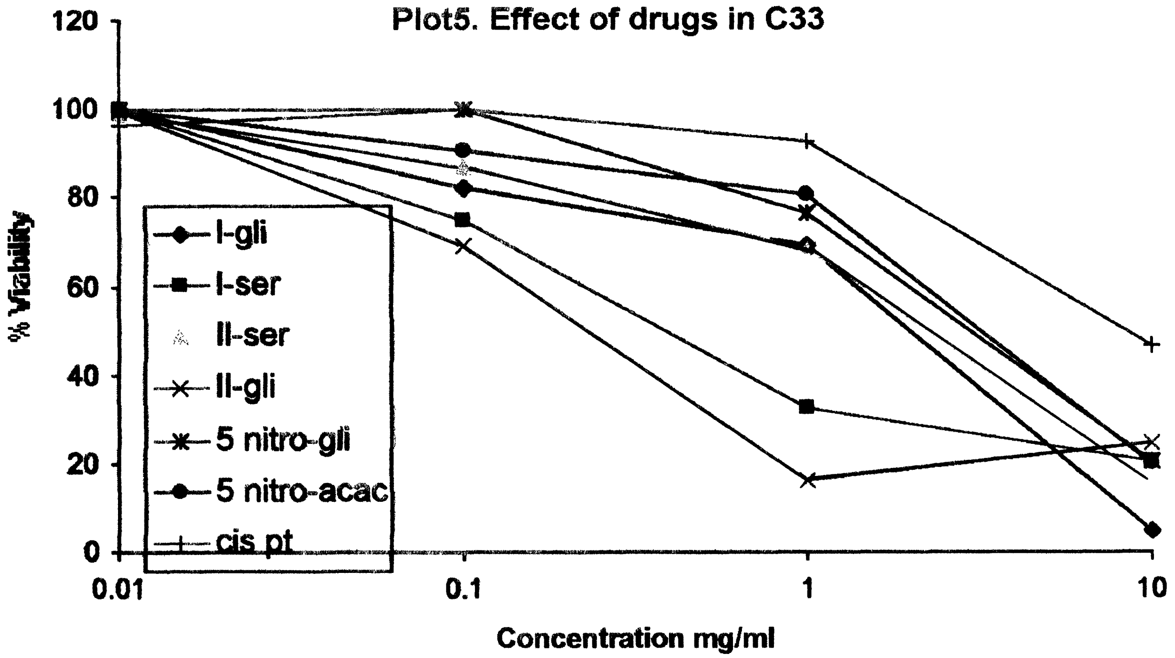

Plot5. Effect of drugs in C33

120

100

80

60

40

20

0

% Viability

I-gli

I-ser

II-ser

II-gli

5 nitro-gli

5 nitro-acac cis pt

0.01

0.1

1

10

Concentration mg/ml

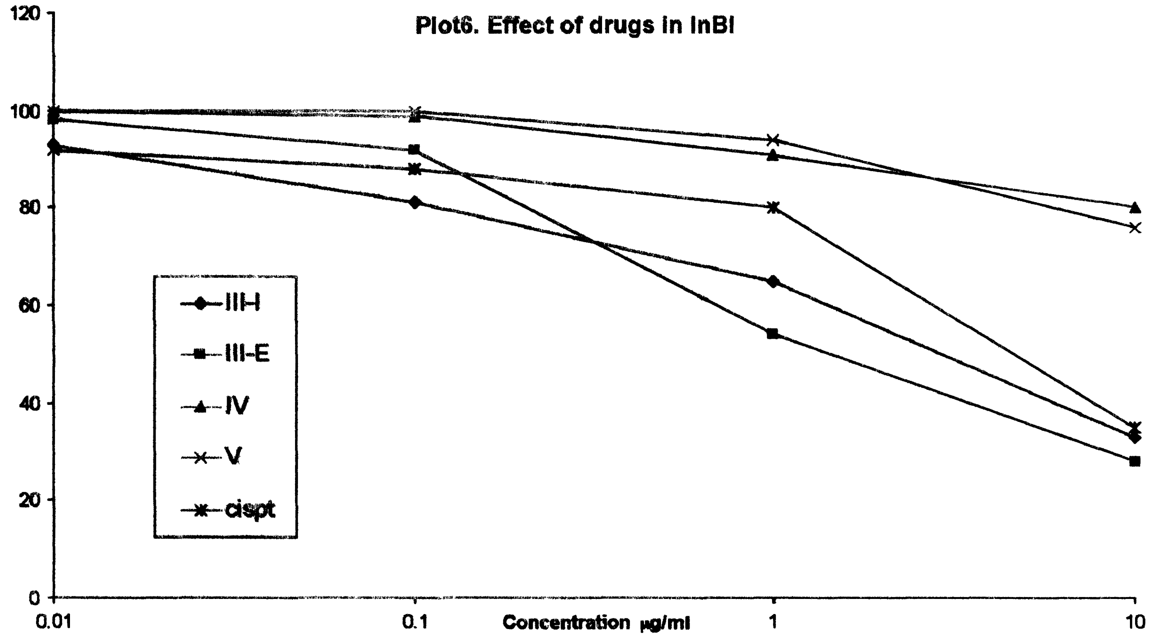

Plot6. Effect of drugs in InBl

120

100

80

60

40

20

0

III-H

III-E

IV

V

cispt

0.01

0.1

Concentration μg/ml

1

10

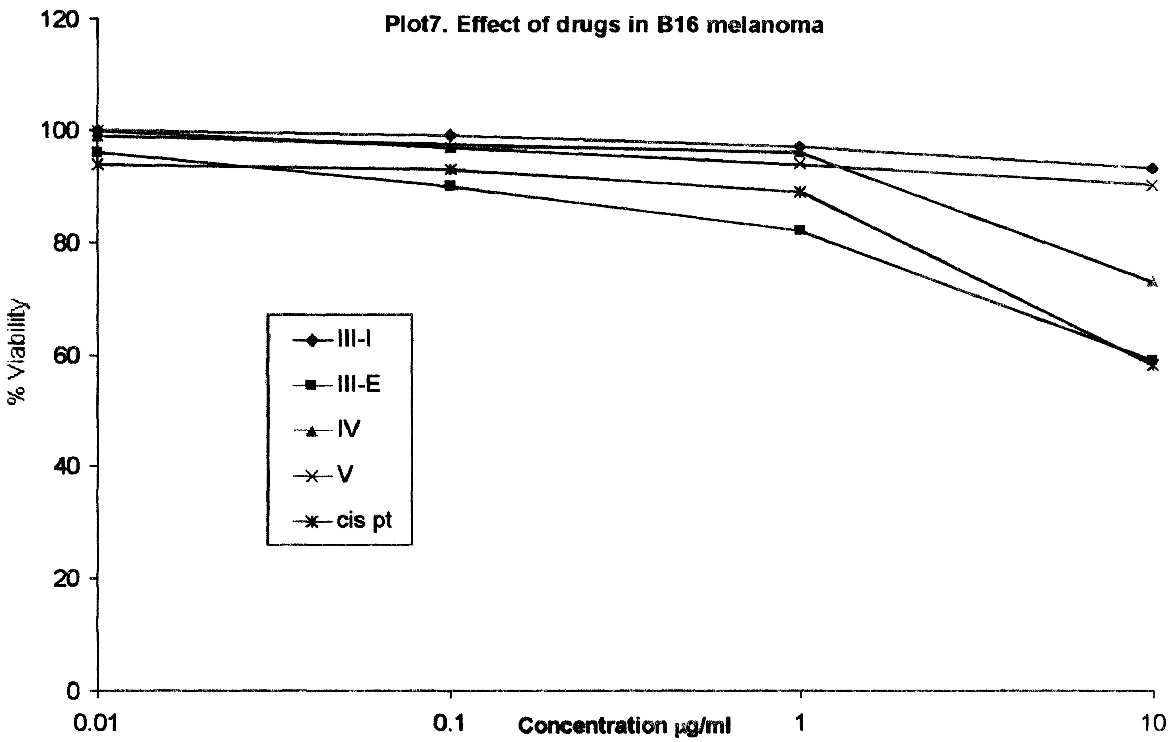

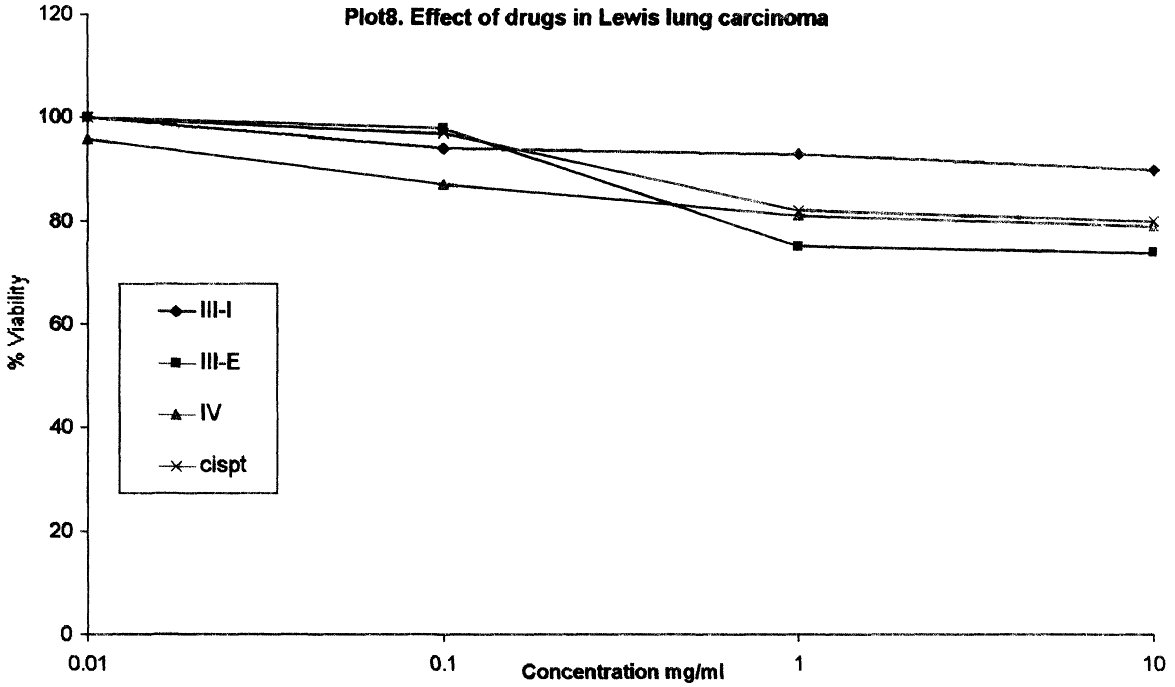

### CONCLUSIONS

At the end of the experiments we conclude that:

- The Casiopeínas showed antineoplastic activity in all the different human and murine cell lines, however, this activity is higher for the cervic-uterine human tumoral cell lines.
- The Casiopeínas with symmetric phenanthrolines, especially with substituents at 4, 7 positions showed more activity than those with substitution in 5 position. Regarding to the O-O donor, the higher activity was showed in those Casiopeínas with glycine.

-The antineoplastic activity of the Casiopeínas and Cisplatin is low for highly mestastatic cell lines like Lewis Lung Carcinoma and B16 Melanoma.
-The monochelate copper complexes shown a very low activity comared with those of the Casiopeinas, these results clearly indicated that the activity of Casiopeinas is due to the whole molecule.
-Casiopeínas keep being a promising resource for the treatment of neoplastic diseases, because have shown higher activity than the control drug (Cisplatin).

**ACKNOWLEDGMENTS**

We thank the program PAPIIT IN100893, IN20793, IN20996 and Conacyt 25510N for financial support

**REFERENCES**

®-Trade Mark :Casiopeína. Reg. 407543 SECOFI (1992)
1.- Lena Ruiz-Azuara. Procedimiento para la obtención de complejos metálicos como agentes anticancerígenos. Tipo I. Patente de invención en trámite, SECOFI 18801. P. I. (1990).Patente, Enero 26, (1994) no. 172967.
2.- Lena Ruiz-Azuara. Procedimiento para la obtención de complejos metálicos como agentes anticancerígenos. Tipo II. Patente de invención en trámite, SECOFI 18802. P. I. (1990).Patente, Dic.9 (1993) no. 172248.
3.- Lena Ruiz-Azuara. Process to obtain new mixed copper aminoacidatecomplexes from phenylatephenanthroline to be used as anticancerigenic agents.
U. S. Patent application serial No. 07/628,843., Ap 21 (1992) Number 5, 107, 005.
U. S Patent Re35, 458, Feb. 18 (1997)
4.- Lena Ruiz-Azuara. Process to obtain new mixed copper aminoacidate from methylate phenantrholine complexes to be used as anticancerigenic agents.
U. S. Patent application serial No. 07/628,628., (1992), Pat. No. 5,576,326. Nov. 19 (1996)
5.- Rafael-MorenoEsparza, Elies Molins, José Luis Briansó, Lena Ruiz-Ramírez and Rocío Redón. "Aqua (1, 10- phenanthroline)(L-serinato)copper (II) Nitrate". *Acta Crystallographica* (1995) C51, 1505-1508.
6.- A. Tovar-Tovar, L. Ruiz Ramírez, A. Campero, A. Romerosa, R. Moreno-Esparza, M.J. Rosalez-Hoz, Structural and reactivity studies on 4,4-dimethyl-2,2-bipyridine,acethylacetonate copper (II) nitrate (Casiopeina III-I) with DNA constituents, by UV-vis and EPR Techniques., *J. of Inorganic Biochemistry*, sent for pubication.
7.- Ruiz-Ramírez L., Gracia-Mora I., Moreno-Esparza R., Díaz D., Gasque L., Huerta L., Mayet L., Lomelí C., "The antitumor activity of several transition metal complexes", *Journal of Inorganic Biochemistry*, 43, 2-3 (1991) 615.
8.- Ferrer Sueta G., Ruiz-Ramírez L., Radi R., Ternary copper complexes and manganese (III) tetrakis (4-benzoic acid)(porphirin) catalyze peroxynitrite-dependent nitration of aromatics., *J. of Chemical Research in Toxicology*, (1997) 10, 12, 1338-13344.
9.- A. de Vizcaya-Ruiz, A. Rivero- Muller, L. Ruiz –Ramírez, G. E. N. Kass, L.R. Kelland, R. M. Orr and M. Dobrota, Induction of Apoptosis by a Novel Copper-based Anticancer Compound- Casiopeina II In L1210 and CH1 Cells. *Toxicology in Vitro*. (2000) 14, 1-5.
10.- Ruiz-Ramírez L., Gracia I., de la Rosa M:E., Sumano H., Gómez C., Arenas F.,Gómez E. Pimentel E.and Cruces M., "Cytostatic, Mutagenic, Antineoplastic activities and prelimina toxicity of copper (II) new drugs: Casiopeinas I, II and III.", *J.of Inorganic Biochemistry*,51 (1-2) (1993) 406.
11.- Geran, R.I., Greenberg N.H., MacDonald, M.M., Schumacher, A.M., and Abbott, B.J., Protocols for sreening chemical agents and natural products against animal tumors and other biological systems. ·3rd. ed., *Cancer Chemother. Rep. Part III*, 3, 47-52 (1972).
12.- American Type. Culture Collection. Cataloge of Cell Lines and Hibridomas. 7th edition, 1992.
13.- Skehan, P., Storeng, R., New Colorimetric Cytotoxity Assay for Antiancer Drug Screening. Journal of the National Cancer Institute 82:1107-1112:1990.
14.- Rubinstein, L. V., Comparison of in vitro Anticancer Drug Screening Data Generated with a Tetrazolium Assay Versus a Protein Assay Against a Diverse Panel Of Human Tumor Cell Lines. Journal of the National Cancer Institute 82:1113-1118:1990.
15.- Alley M.C., Scudiero D.A, Monks A., *et al.* Feasibility of drug sreening with panels of human tumor cell lines using a microculture tetrazolium assay, *Cancer Research* 1998; 48:598.

